# Novel mitochondrial derived Nuclear Excisosome degrades nuclei during differentiation of prosimian Galago (bush baby) monkey lenses

**DOI:** 10.1371/journal.pone.0241631

**Published:** 2020-11-12

**Authors:** M. Joseph Costello, Kurt O. Gilliland, Ashik Mohamed, Kevin L. Schey, Sönke Johnsen, Lisa A. Brennan, Marc Kantorow

**Affiliations:** 1 Department of Cell Biology and Physiology, University of North Carolina, Chapel Hill, NC, United States of America; 2 Ophthalmic Biophysics, L V Prasad Eye Institute, Hyderabad, Telangana, India; 3 Biochemistry Department, Vanderbilt University, Nashville, TN, United States of America; 4 Biology Department, Duke University, Durham, NC, United States of America; 5 Department of Biomedical Science, Florida Atlantic University, Boca Raton, FL, United States of America; Washington University in Saint Louis School of Medicine, UNITED STATES

## Abstract

The unique cellular organization and transparent function of the ocular lens depend on the continuous differentiation of immature epithelial cells on the lens anterior surface into mature elongated fiber cells within the lens core. A ubiquitous event during lens differentiation is the complete elimination of organelles required for mature lens fiber cell structure and transparency. Distinct pathways have been identified to mediate the elimination of non-nuclear organelles and nuclei. Recently, we reported the discovery of a unique structure in developing fiber cells of the chick embryo lens, called the Nuclear Excisosome, that is intractably associated with degrading nuclei during lens fiber cell differentiation. In the chick lens, the Nuclear Excisosome is derived from projections of adjacent cells contacting the nuclear envelope during nuclear elimination. Here, we demonstrate that, in contrast to the avian model, Nuclear Excisosomes in a primate model, Galago (bush baby) monkeys, are derived through the recruitment of mitochondria to form unique linear assemblies that define a novel primate Nuclear Excisosome. Four lenses from three monkeys aged 2–5 years were fixed in formalin, followed by paraformaldehyde, then processed for Airyscan confocal microscopy or transmission electron microscopy. For confocal imaging, fluorescent dyes labelled membranes, carbohydrate in the extracellular space, filamentous actin and nuclei. Fiber cells from Galago lenses typically displayed prominent linear structures within the cytoplasm with a distinctive cross-section of four membranes and lengths up to 30 μm. The outer membranes of these linear structures were observed to attach to the outer nuclear envelope membrane to initiate degradation near the organelle-free zone. The origin of these unique structures was mitochondria in the equatorial epithelium (not from plasma membranes of adjacent cells as in the chick embryo model). Early changes in mitochondria appeared to be the collapse of the cristae and modification of one side of the mitochondrial outer membrane to promote accumulation of protein in a dense cluster. As a mitochondrion surrounded the dense protein cluster, an outer mitochondrial membrane enclosed the protein to form a core and another outer mitochondrial membrane formed the outermost layer. The paired membranes of irregular texture between the inner core membrane and the outer limiting membrane appeared to be derived from modified mitochondrial cristae. Several mitochondria were involved in the formation and maturation of these unique complexes that apparently migrated around the fulcrum into the cytoplasm of nascent fiber cells where they were stabilized until the nuclear degradation was initiated. Thus, unlike in the chick embryo, the Galago lenses degraded nuclear envelopes with a Nuclear Excisosome derived from multiple mitochondria in the epithelium that formed novel linear assemblies in developing fiber cells. These findings suggest that recruitment of distinct structures is required for Nuclear Excisosome formation in different species.

## Introduction

The mature transparent structure of the ocular lens is dependent on an elaborate differentiation program that converts cuboidal epithelial cells at the lens surface into elongated fiber cells in the lens core. A hallmark event of the lens differentiation program is the degradation of all membranous organelles, including Golgi, endoplasmic reticulum (ER), mitochondria and nuclei, to generate a lens core without intracellular light scattering centers. The phenomenon of organelle degradation to create the organelle-free zone (OFZ) was recognized over 120 years ago by C. Rabl [1] and has been studied extensively in a variety of species as discussed in several reviews [2–5]. An important advance was the recognition that autophagy played a key role in degradation of most of the organelles except for the nucleus [6, 7], and recent studies demonstrated that the elimination of non-nuclear organelles during lens fiber cell differentiation is mediated through the actions of the mitophagy-associated protein BNIP3L [8].

In contrast to the elimination of non-nuclear organelles, the elimination of nuclei during lens differentiation is mediated by a distinct pathway characterized by the formation of a novel structure called the Nuclear Excisosome [9]. In the chick embryonic lens, the Nuclear Excisosome (NE) is formed by numerous finger-like projections arising from adjacent cells and extending to the nuclear outer membrane [9]. The extensions from adjacent cells looked like typical interlocking devices, such as ball-and-socket interdigitations, which were initiated by a coat of clathrin and propelled by an internal network of actin [10]. These intercellular projections were present in most vertebrate lenses and were quite short (about 0.5 μm) and the clathrin coat came off very early after formation. In the chick embryo model, the projections were significantly longer (up to 3 μm) and retained the clathrin coat even after making contact with the outer membrane of the nuclear envelope [9]. Importantly, at the contact sites, appendages were found that were unlike any known organelle and could be interpreted as degrading the outer and inner nuclear envelope membranes.

In an effort to uncover analogous nuclear degradation structures in higher primates, lenses from humans and macaque monkeys were examined in detail (Costello MJ, et al., Invest Ophthalmol Vis Sci. 2017;58:ARVO E-Abstract 1213; Costello MJ, et al., Invest Ophthalmol Vis Sci. 2019;60:ARVO E-Abstract 2233). As expected, these lenses displayed numerous ball-and-socket interdigitations with early forms clearly initiated with clathrin coats and later forms displaying an internal actin network consistent with earlier work [10]. However, none of these interdigitations were as long as in the chick embryo, nor did they contact the nuclear envelope or appear to participate in nuclear degradation. Instead, these higher primates displayed unique structures around nuclei that included numerous clustered vesicles around nuclei in human lenses and very large multilayerd objects, first noted by S. Bassnett [11], in macaque monkey lenses that were similar to smooth ER derived intracellular inclusions in other tissues [12]. Confocal images of higher primate lenses clearly displayed large objects associated with degrading nuclei that are likely to be components of the NE, although the cell biological and molecular interpretation have not yet been resolved. The characterization of nuclear degradation in these higher primates is complicated by two factors: the zone of the lens where organelle elimination takes place (the remodelling zone) is characterized by extensive cellular disruption and reorganization at the same location that final nuclear degradation is occurring [13] and the degradation process is relatively rapid with intact nuclei reduced to nuclear fragments within a few cell layers in a band about 50 μm thick. By contrast, the chick model was embryonic with relatively minor cellular changes in the remodelling zone and the early developmental stage provided nuclear degradation over more than 600 μm radial distance where many intermediate stages of nuclear breakdown could be observed [9].

To further explore the apparent species specificity associated with NE formation and to overcome some of the limitations presented in the higher primate lenses, we examined lenses from the lesser Galago (bush baby) monkey. Although only a limited number of specimens were available, this species provided some significant advantages in the study of lens ultrastructure and organelle degradation. Specifically, the nuclear degradation occurred over a radial distance of greater than 300 μm and the cellular modifications within the remodelling zone were relatively minor compared to the higher primates. The lenses were large within large eyes, consistent with the nocturnal life in variable habitat in sub-Saharan Africa where extensive non-endangered populations exist and are known for their agility and capability to capture insects with the skill and precision of bats without the use of sonar.

Surprisingly, the initial examination of the outer cortex of Galago lenses did not reveal any finger-like filapodial extensions from adjacent cells, as seen in chick embryo lenses [9], although numerous typical ball-and-socket interdigitations were present. Instead, large and prominent linear objects in the fiber cell cytoplasm appeared to be the active component in nuclear degradation. The cross-section of the linear objects contained distinctive morphological features that allowed the source of the objects to be identified as the equatorial epithelium and their genesis to be from modified and fused mitochondria. The long distance from the formation in the epithelium to the site of action near the OFZ means that the structures must be stabilized within the fiber cell cytoplasm until they are triggered to attack and degrade nuclear envelopes. The data are presented here in the order of (a) identification of linear objects at the site of activation on outer nuclear envelope membranes, (b) characterization of the unique morphology of the linear structures, (c) identification of the initiation of degradation induced by the linear objects and (d) determination of the role of mitochondria in the formation and maturation within the lens epithelium. These data support the conclusion that distinct structures govern the elimination of nuclei during lens fiber cell differentiation in avian and primate lenses. The data also point to the requirement for novel mitochondrial assemblies for Galago NE formation and nuclear elimination to form mature lens fiber cells.

## Materials and methods

Lenses from Galago (bush baby) monkeys were obtained from Vanderbilt University following dissection from eyes provided by the Vanderbilt Primate Center through a tissue sharing protocol following euthanasia of animals with a high dose of sodium pentobarbital (120 mg/kg) under a project approved by the Vanderbilt University Institutional Animal Care and Use Committee not related to the lens studies presented here. All experiments adhered to the Association for Research in Vision and Ophthalmology Statement for the Use of Animals in Ophthalmic and Vision Research. Four extracted lenses from three animals aged 2–5 years were fixed initially in 10% neutral buffered formalin (number 15740, EMS, Hatfield, PA) at Vanderbilt following the procedures described previously [14] and then express shipped to the University of North Carolina (UNC). After 24 hours fixation in formalin, the lenses were fixed for 48 hours in freshly prepared 4% paraformaldehyde (number 19208, EMS, Hatfield, PA), then rinsed and stored in 0.1 M cacodylate buffer at 4°C until Vibratome processing [14].

Confocal specimens were prepared from 120 μm thick Vibratome sections transferred to Tyrode’s buffer (Sigma-Aldrich, T2397), stained either for actin or for membranes at room temperature with rotation in the dark. For actin staining, Vibratome sections were first stained with Wheat Germ Agglutinin (WGA, ThermoFisher conjugated with Alexa 488, W11261) about 1 mg/ml stock of lyophilized WGA in buffer, washed twice, followed by permeabilization with 0.2% Triton X-100 (ThermoFisher, 28313) for 15 min, washed twice, followed by staining with Phalloidin-Alexa 568 (ThermoFisher, A12380) for 20 min and washed twice. For membrane staining, the carbocyanine dye DiI was employed (ThermoFisher, Vybrant CM-DiI, V22888) for 15 minutes in 50% ethanol, washed twice for 5 min in buffer. After either staining protocol, sections were stained for nuclei with DAPI (ThermoFisher, D1306) for 10 minutes followed by washing twice for 5 minutes in buffer. Stained sections were mounted with a water soluble medium (Shur Mount 17992, EMS, Hatfield, PA) beneath a 17 μm thick 1.5 glass coverslip and sealed with a stainless steel annular washer 120 μm thick (Phoenix Specialty, Bamberg, SC) in the following sequence: an annular washer was glued (Loctite Glass Super Glue) to a 22 mm square coverslip; a stained section was placed on the coverslip, water wicked away and covered with mounting medium just sufficient to fill the volume; glue was placed on top of the washer and a glass slide was placed and weighed to seal the assembly. Confocal images were obtained with a Zeiss LSM 880 with Airyscan (Carl Zeiss Microscopy, Jena, Germany), using a Zeiss 63x oil lens, numerical aperture 1.4, as the primary objective. Excitation for DiI and actin dyes was with a diode laser at 561 nm; for WGA dye at 488 nm and DAPI at 405 nm. The detector was the new Airyscan design [15] operated in the super-resolution mode that essentially uses 32 pinhole detectors, which increases resolution to about 140 nm in the x, y and z by capturing and analyzing more image data at the back focal plane of the objective.

Fixed lenses were stored in 0.1M cacodylate buffer until processing for transmission electron microscopy (TEM) as described previously [16]. Briefly, fixed lenses were Vibratome sectioned to 120 μm thickness, immersion fixed in 2.5% glutaraldehyde, 2% paraformaldehyde, 1% tannic acid in 0.1M cacodylate buffer, pH 7.2 for 12 hours. After washing, fixed thick sections were stained in 0.5% osmium tetroxide at 4°C for 1 hour followed by en bloc staining in 2% uranyl acetate for 1 hour in 50% ethanol. Sections were dehydrated in an ethanol series, infused with propylene oxide and embedded in epoxy resin (Epon 812, EMS, Hatfield, PA). Thin sections 70 nm thick near the equatorial plane from the capsule to the lens center were cut with an ultramicrotome (Leica Ultracut UC7, Leica Microsystems, Vienna, Austria) using a Diatome diamond knife (EMS, Hatfield, PA). Grids (hexagonal thin bar, 600 mesh) stained with uranyl acetate and lead citrate were examined for structures involved in nuclear breakdown using a ThermoFisher (FEI) Tecnai T12 G2 TEM (Hillsboro, OR) operated at 80–120 kV equipped with a high resolution 1k x 1k CCD camera (Model 794) or a Rio16 4k x 4k CMOS camera (Gatan, Pleasanton, CA).

## Results

### A. Unique linear structures within Galago fiber cell cytoplasm

**Fig 1:**
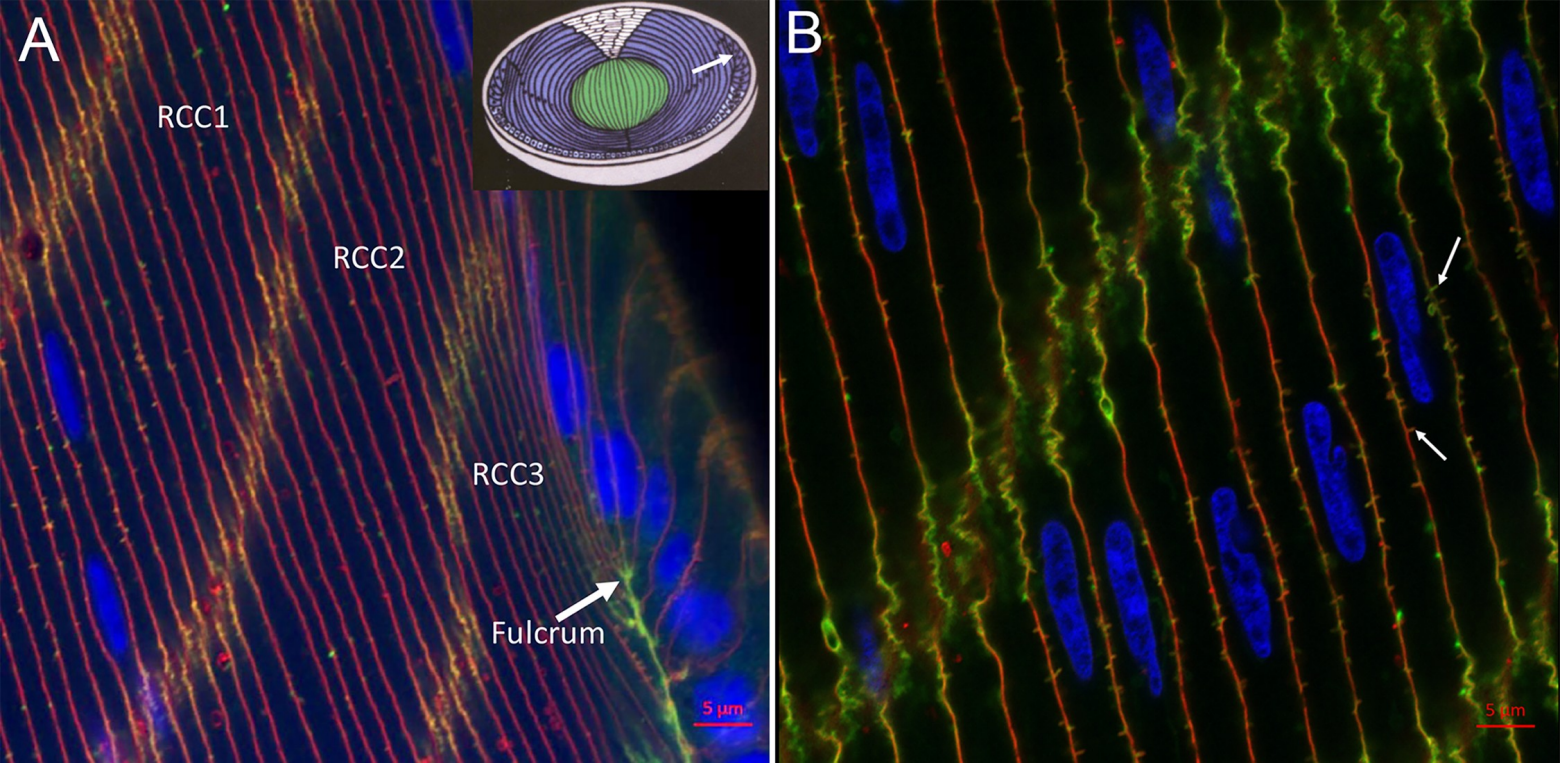
Airyscan confocal at the fulcrum and bow zone. Cells are outlined with WGA mainly labeling carbohydrate within extracellular spaces (red). Filamentous cytoplasmic actin is labeled with phalloidin (green). As in all confocal images, nuclei are stained with DAPI (blue). The lipid bilayers of membranes are not labelled in this preparation. (A) The transition from epithelial cell to fiber cell is shown at the fulcrum where an enhanced adherens junction occurs as indicated by the intense actin staining (green). Radial cell columns (RCC1-3) are separated by interlocking short sides of hexagonal fiber cells where adherens junctions dominate. Inset: Diagram of early fetal development of a mammalian lens with the fulcrum indicated (arrow) and the beginning of the OFZ where the nuclei (dark spots) have been eliminated. (Reprinted from: Kuszak JR, Costello MJ. Structure of the vertebrate lens. In: Lovicu FJ, Robinson ML, editors. Development of the Ocular Lens. Cambridge, UK: Cambridge Univ. Press; 2004. pp. 71–118.) (B) Bow region about 40 μm deep showing about 9 nuclei (blue) among fiber cells clearly outlined with WGA (red) without direct labeling of the membrane bilayers. Tiny blips on the broad faces are mostly ball-and-socket interdigitations consisting of a membrane pair extending about 0.5 μm into adjacent cells. The extracellular space between membranes is stained red with WGA and the actin in the core of each ball-and-socket is stained green; an example of each label is marked (arrows). A gradual color shift from red to green through yellow and orange can be seen by focusing the microscope at slightly different levels, which allows emphasis of each dye separated by the unstained membrane bilayer. This image emphasizes the accurate staining of actin in the absence of long filapodia-like extensions as seen in chick embryo lenses.

The key transition point in the differentiation of epithelial cells into fiber cells is captured in super-resolution confocal images (Fig 1). The four nuclei in the epithelium represent the region that is proposed to be the primary site of production of new NE components (Fig 1A, blue, right side). The last epithelial cell in this cluster appears to be anchored at the fulcrum (Fig 1A, arrow; inset, white arrow) by a strong adherens junction supported by filamentous actin (green) on the cytoplasmic sides of the junction. The actin staining is also prevalent within the interfaces between radial cell columns (Fig 1A, RCC1-3) where adherens junctions exist [17, 18]. The yellow to orange color along the interfaces is due to the presence of WGA (red) that labels glycoproteins and glycolipids extending into the extracellular space. The red fluorescence is highest along the broad faces of fiber cells and between epithelial cells where the amount of filamentous actin is minimal. A more detailed view of actin labelling is visible in a view slightly deeper where portions of at least 9 nuclei from the bow region are visible (Fig 1B, blue). The intense staining of actin along the interface between two RCCs (green) is distinctive and WGA staining highlights the extracellular space along the broad faces (red) as well as outlining the fiber cells (Fig 1B). The numerous tiny blips, mainly along the broad faces of fiber cells, are ball-and-socket interdigitations about 0.5 μm long (Fig 1B, white arrows). These display either red or green, or a combination, consistent with the known structure of a projection of a pair of membranes separated by a constant extracellular space and containing a core of actin [10]. Depending on how the optical section intersects these doubly labelled structures (on either side of the lipid bilayer membrane but not the bilayer membrane itself), the fluorescence of the dyes are emphasized differently. The most important conclusion from this image is that none of the projections extend far from the membrane surfaces in filopodial-like projections with actin cores in contrast to the chick embryo lenses.

**Fig 2:**
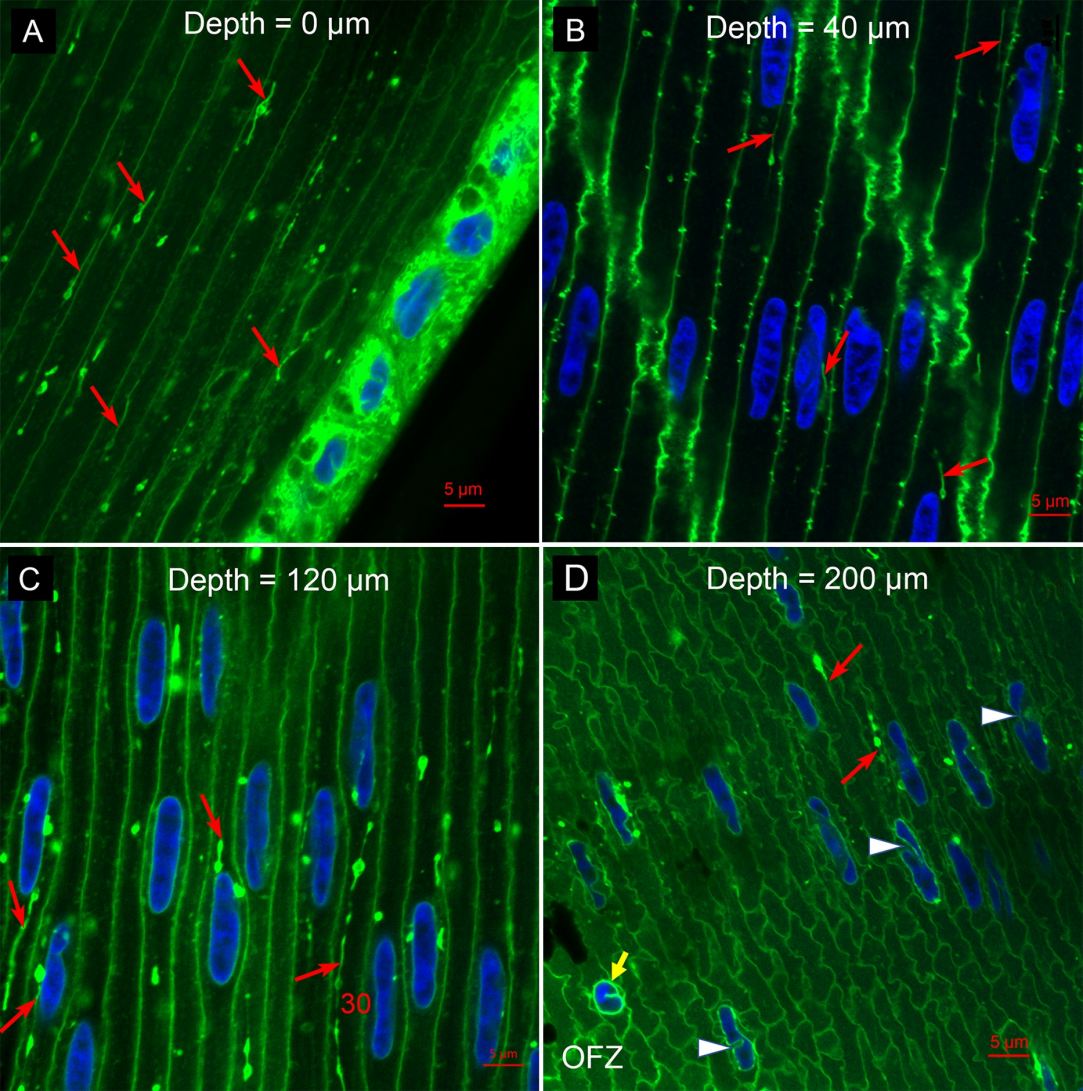
Membrane staining revels novel linear structures. (A) Epithelium and young fiber cells. At the epithelial-fiber cell interface (0 μm depth), the border between adjacent fiber cells is clearly delineated. There are numerous short and long (up to 10 μm) linear objects that appear to be contained within the fiber cell cytoplasm (red arrows). These are not typically found in fiber cells of other species. The diameter of linear segments is about 0.5–1 μm and occasional bulges are slightly larger. (B) In the bow zone, more than 10 nuclei (blue) are between and separated from broad cell interfaces decorated with small blips of ball-and-socket interdigitations typically found in fiber cells. Atypical are the linear structures (red arrows) that are closely associated with or in contact with nuclei. (C) At a depth where nuclear breakdown has begun (120 μm), the linear structures are prominent and in contact with nuclei (red arrows). These linear structures can be quite long, up to 30 μm (red number). (D) At a greater depth (200 μm), where some nuclear breakdown is nearly complete (yellow arrow), the linear structures are still present (red arrows). In this region, the cell shapes can be irregular and the nuclei extensively deformed (white arrowheads).

When the bilayer component of membranes is stained with DiI, the internal structure of fiber cells in the outer cortex reveals the presence of numerous linear structures (Fig 2). Close to the epithelium, these linear structures appear to be within the fiber cell cytoplasm and not derived from or bound to the plasma membranes (Fig 2A, red arrows). The lengths vary from about 5 to 10 μm in this image with diameters of the rod sections of about 0.5 to 1 μm and frequent bulges of over 2 μm diameter. Such linear structures are not commonly seen in fiber cells of other species. The close proximity of the linear structures to the epithelium indicates that they are present in young fiber cells far from the OFZ where nuclear degradation is occurring. At a slightly greater depth into the lens of 40 μm, the linear structures appear to be associated with intact nuclei of the bow zone (Fig 2B, red arrows). As in Fig 1B, the small blips on the long faces of fiber cells are most likely ball-and-socket interdigitations, in this case with the membrane bilayers stained. A comparison with the actin stained images in Fig 1 suggests that the novel linear structures do not contain actin. At a depth of about 120 μm where nuclear degradation is beginning, the linear structures are prominent and closely associated with the surfaces of nuclei (Fig 2C). In this view, one of the linear structures is over 30 μm long with a beads-on-a-string appearance (Fig 2C, next to the number 30). At 200 μm depth where some nuclear degradation is obvious, the linear structures are still present (Fig 2D, red arrows) along with distorted nuclei (white arrowheads) and nuclear fragments (yellow arrow) next to the OFZ. Fiber cells are distorted and irregular following changes in the remodelling zone of primate lenses [13]. The images in Fig 2 are representative of the lens outer cortex where the degradation of nuclei is clearly correlated with and involves linear structures as potential components of the NE in Galago lenses.

### B. Thin section TEM reveals the internal structure of the Galago NE

**Fig 3:**
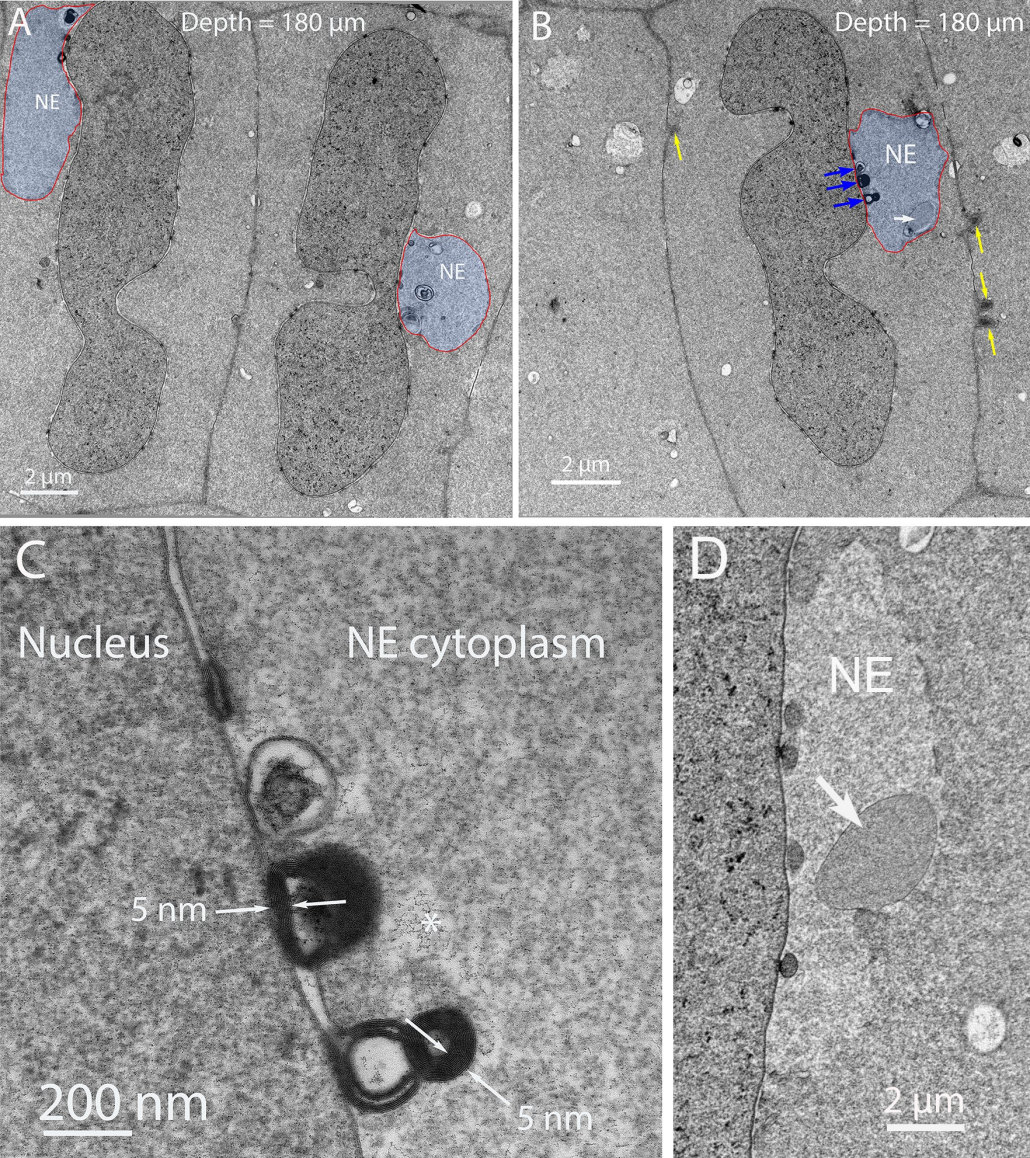
Thin section transmission electron microscopy of nuclear degradation in Galago lenses. (A) Two nuclei that are deformed indicate that degradation is in progress. Each has an object identified as a component of the Nuclear Excisosome (NE) with a faint outline (red line) and a low-density region (blue) that contains components of the degradation machinery and products of degradation including modified proteins and multilamellar lipids. (B) Another example of a nucleus with an adherent NE containing multilamaellar lipid (blue arrows) and a remnant of the initiation complex (white arrow). Also noted are four ball-and-socket structures that typically are 0.5 μm long paired membrane projections (yellow arrows). (C) High magnification of the multilamellar lipid for which the 5 nm spacing is evident. This is a product of the degradation as are the modified proteins inside the NE, most likely including the lacy filamentous clusters (asterisk). (D) An example from a different nucleus shows distinctive variations in staining density across the NE. The dense nucleus on the left is separated by the dark inner nuclear envelope membrane and four circular profiles of early membrane breakdown from the lighter zone of the NE, which is outlined by an irregular border adjacent to the darker fiber cell cytoplasm. A remnant of the degradation complex (white arrow) stains uniformly and is surrounded by single membrane as a fine dark line.

Low magnification overviews of nuclei in the zone where degradation has begun shows irregular shapes of nuclei and appendages on the surfaces of nuclear envelopes that correspond to the NE (Fig 3). These micron sized structures are similar in overview appearance to those observed in the chick embryo model [9], except that both the detailed ultrastructure and origin are quite different. Subtle variations of staining density around the nuclei yields a boundary around each proposed NE (Fig 3A, red lines). The interiors contain components of degradation of unknown composition which mainly appear to be proteins based on the similarity of staining to the adjacent protein-rich cytoplasm, although at lower density (Fig 3A, blue highlight). Over 50 such nuclei have been recorded in various stages of degradation of the outer nuclear envelope membrane at depths of roughly 100–200 μm. In some thin sections, it is possible to identify relevant components, such as a smooth nearly circular region within the NE (Fig 3B, white arrow), as well as dense aggregates of lipid from the breakdown of the nuclear envelope (Fig 3B, blue arrows) and enlarged view (Fig 3C). As in the chick embryo model, the final breakdown of the nuclear envelope leaves multilamellar structures with a 5 nm interlamellar spacing, typical of multilayers of lipid bilayers [19]. As suggested for the chick [9], it is thought that the lipid is conserved, redistributed to adjacent plasma membranes and reused. This entire process is coordinated by filapodial-like projections from adjacent cells in the chick that are not present in the Galago lenses. Well defined ball-and-socket structures are present in Galago lenses (Fig 3B, yellow arrows), consistent with the confocal images, but are only about 0.5 μm in length and do not appear to be involved in NE formation or nuclear degradation in the Galago lenses. An example of a NE displaying a distinctive pattern of staining density gives a characteristic view of this unique complex (Fig 3D).

**Fig 4:**
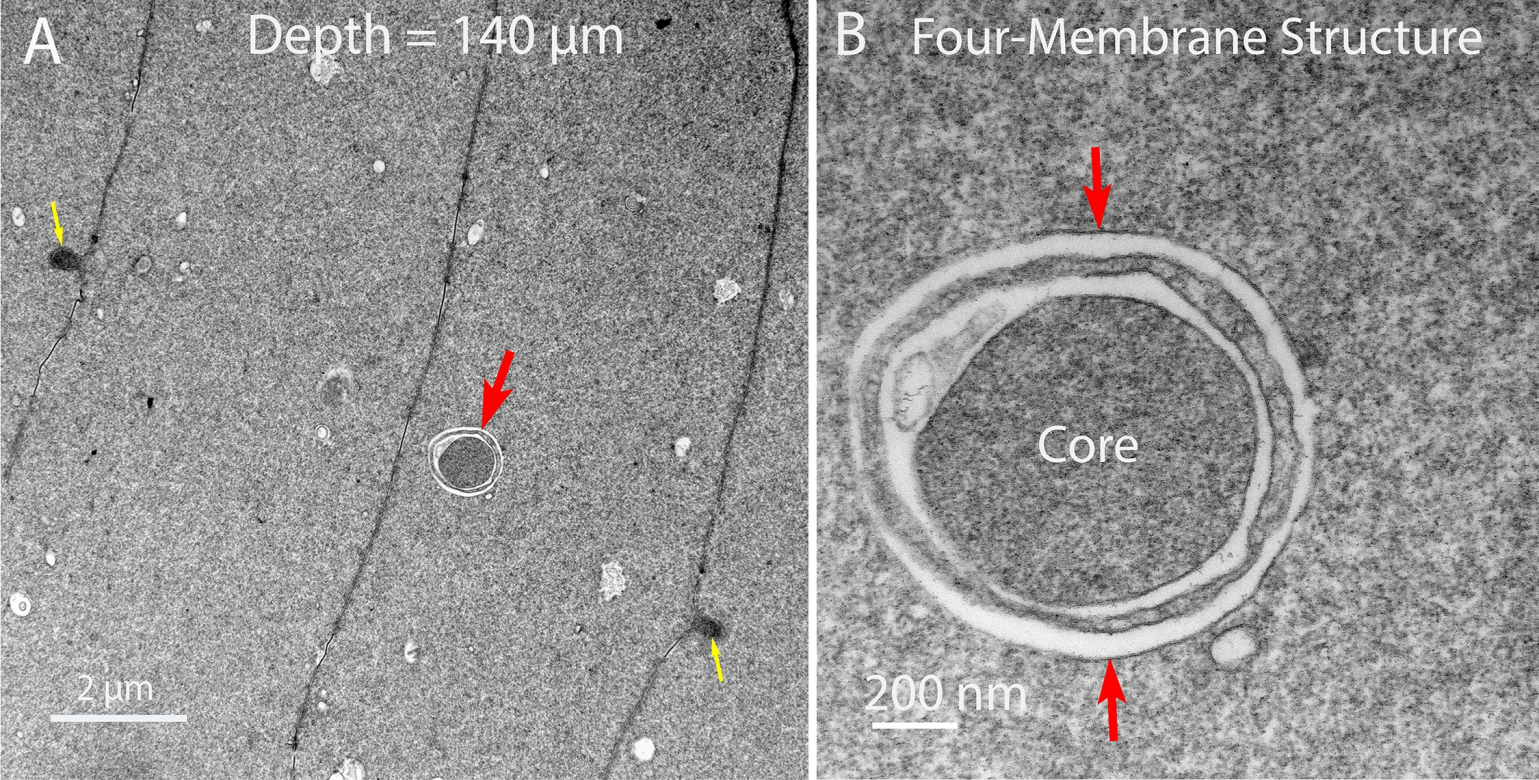
Characterization of the ultrastructure of the linear objects. (A) Cross-sections of the linear structures are a common feature in the TEM images (red arrow). The uniform profile about 1 μm diameter is consistent with the narrow portions of the linear structures in the confocal images. (B) At high magnification the unique profile shows an inner core surrounded by a single membrane, an outer membrane adjacent to the cytoplasm and an intervening membrane pair that is irregular in staining and separation. This is the four-membrane organization that distinguishes this object.

**Fig 5:**
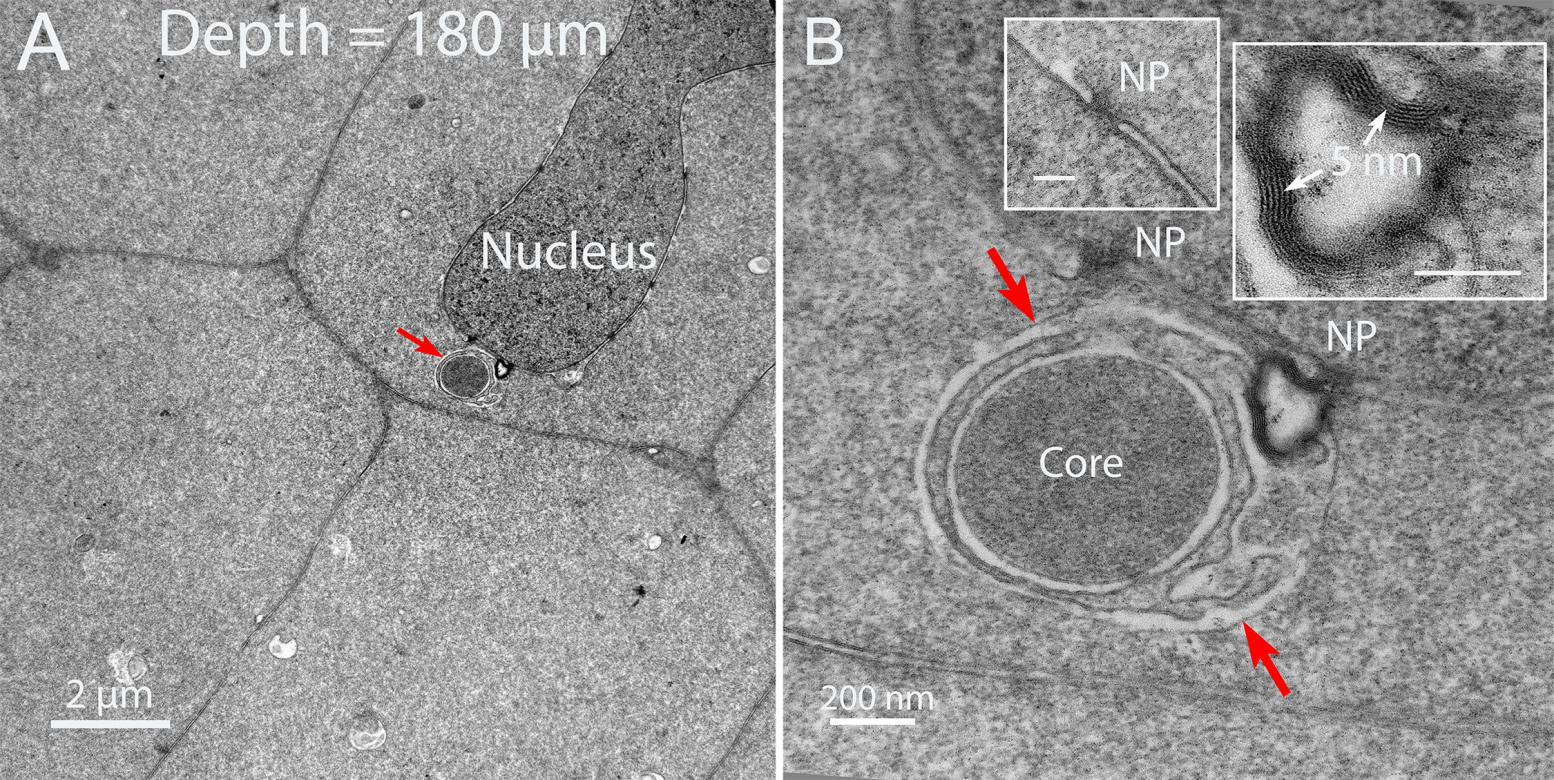
Interaction of the linear object with the nuclear envelope. (A) A four-membrane linear structure is imaged attached to a nucleus at low magnification. (B) At high magnification, the four-membrane profile is remarkably similar to the isolated profile in Fig 4 that is not associated with a nucleus. The interaction with the nucleus includes the fusion of the outer limiting membrane with the outer nuclear envelope membrane and the release of degradation factors from the irregular pair of membranes. The dense regions at the site of contact, although slightly smeared, are most likely nuclear pores (NP) based on the similarity to a neighboring well-preserved NP (inset). The degradation produces multilamellar lipids which display the characteristic 5 nm spacing (inset). Bars in the insets are 100 nm.

**Fig 6:**
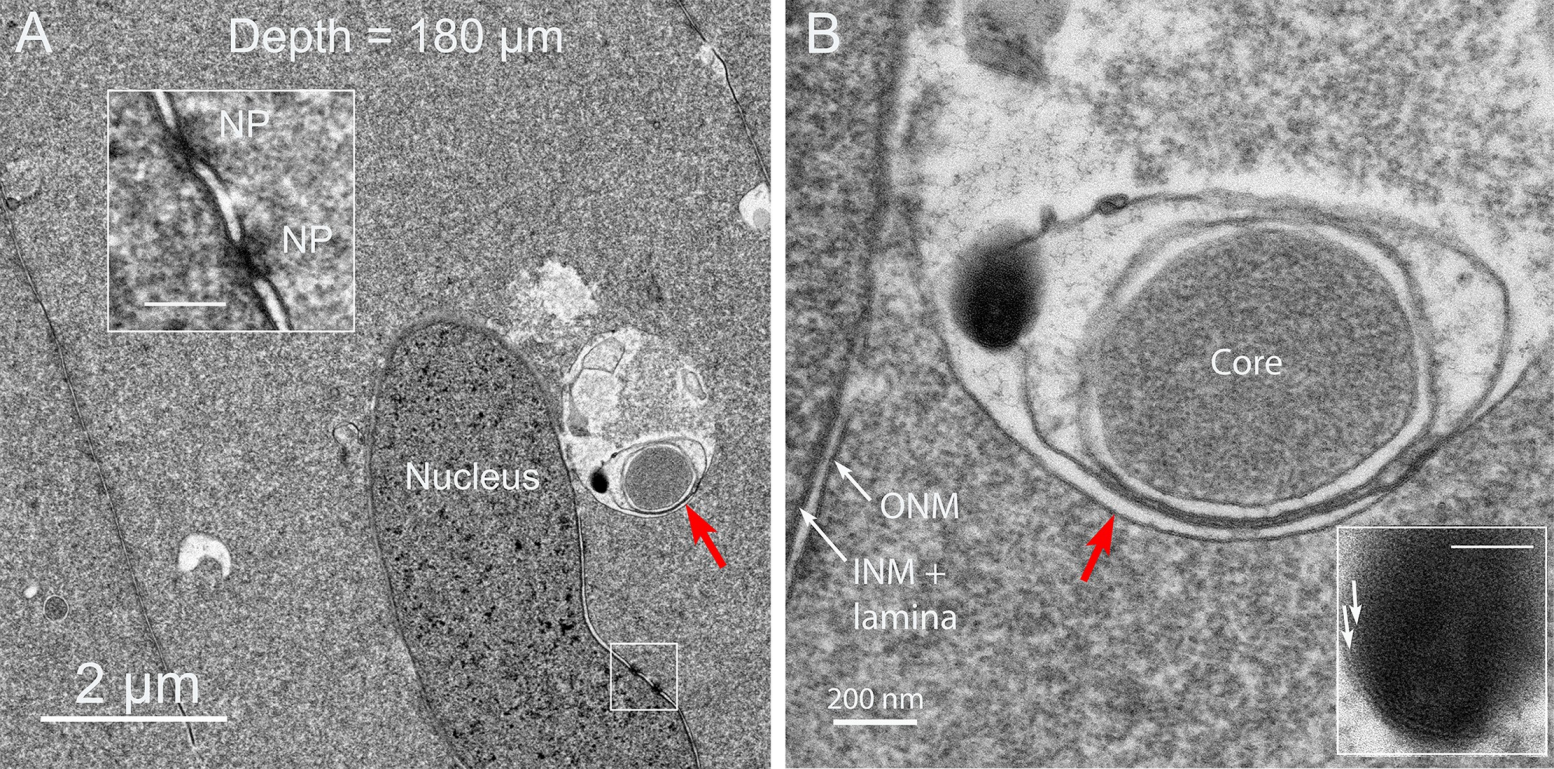
Interaction of a linear object with the nuclear envelope not at a nuclear pore. (A) A second example of a linear four-membrane object initiating degradation at a nucleus. The outer membrane (red arrows) is fused with the outer nuclear envelope membrane close to nuclear pores (NP and inset), although not at the pores as in the first example. (B) At high magnification the outer membrane (red arrow) blends with the outer nuclear membrane and the irregular paired membranes give rise to multilamellar membranes (inset) that could be from the nuclear envelope or the irregular paired membranes. The core is conserved and the outer and inner nuclear membranes (ONM and INM) are clearly displayed. Bars in the insets are 100 nm.

The confocal images of Fig 2 emphasize the cytoplasmic linear objects and the TEM images of Fig 3 identify NE appendages on degrading nuclei. The following images of Figs 4–6 will establish the relationship between these two critical structures. The thin sections are oriented to provide cross-sections of the linear structures. One such cross-section shows a round profile about 1 μm in diameter consistent with the confocal images (Fig 4A, red arrow). At high magnification the unique interior structure is clear (Fig 4B), showing the consistent pattern of an outer membrane (red arrows) that is uniform in thickness and a similar inner membrane around a core of densely stained and remarkably uniform tightly packed protein. The space between the outer and inner membranes is occupied by a pair of membranes that is irregular in staining and separation. This combination produces a four-membrane organelle that is unlike any other organelle in the lens. Thus, in order to move from the core to the cytoplasm, it is necessary to cross four membranes.

These distinctive morphological features are used to identify this object in different settings, including the interaction with nuclei to be degraded (Fig 5A). A four-membrane object is in contact with one end of a nucleus and interacts to initiate the degradation of the outer nuclear envelope membrane (Fig 5B). The outer limiting membrane (red arrows) appears to fuse with the outer nuclear envelope membrane at two nuclear pore complexes (NP). These pores are not in the ideal orientation for imaging, as is the pore further along the nuclear envelope (inset, NP). The core remains intact while the irregular pair of membranes appears to be involved in the nuclear envelope breakdown, which releases multilamellar lipid (inset, 5 nm bilayers). Further details are shown in another example of the interaction of the linear structures with a nucleus (Fig 6A and 6B). The outer membrane of the four-membrane object (red arrow) appears to fuse with the outer nuclear membrane close to nuclear pore complexes (NP and inset) but not exactly at the pores. The irregular pair of membranes has been modified and appears to be part of the degradation process yielding irregular globular and filamentous protein and multilamellar lipid (Fig 6A, inset). The core again remains intact. In the adjacent nuclear envelope membrane, the individual outer and inner nuclear membranes can be clearly identified with sufficient resolution to visualize the nuclear lamina next to the nucleoplasm as a 10 nm thick dense layer, supporting the observation that nuclear degradation can begin in Galago lenses without removal of the nuclear lamina.

### C. Origin of four-membrane structures is the lens epithelium

**Fig 7:**
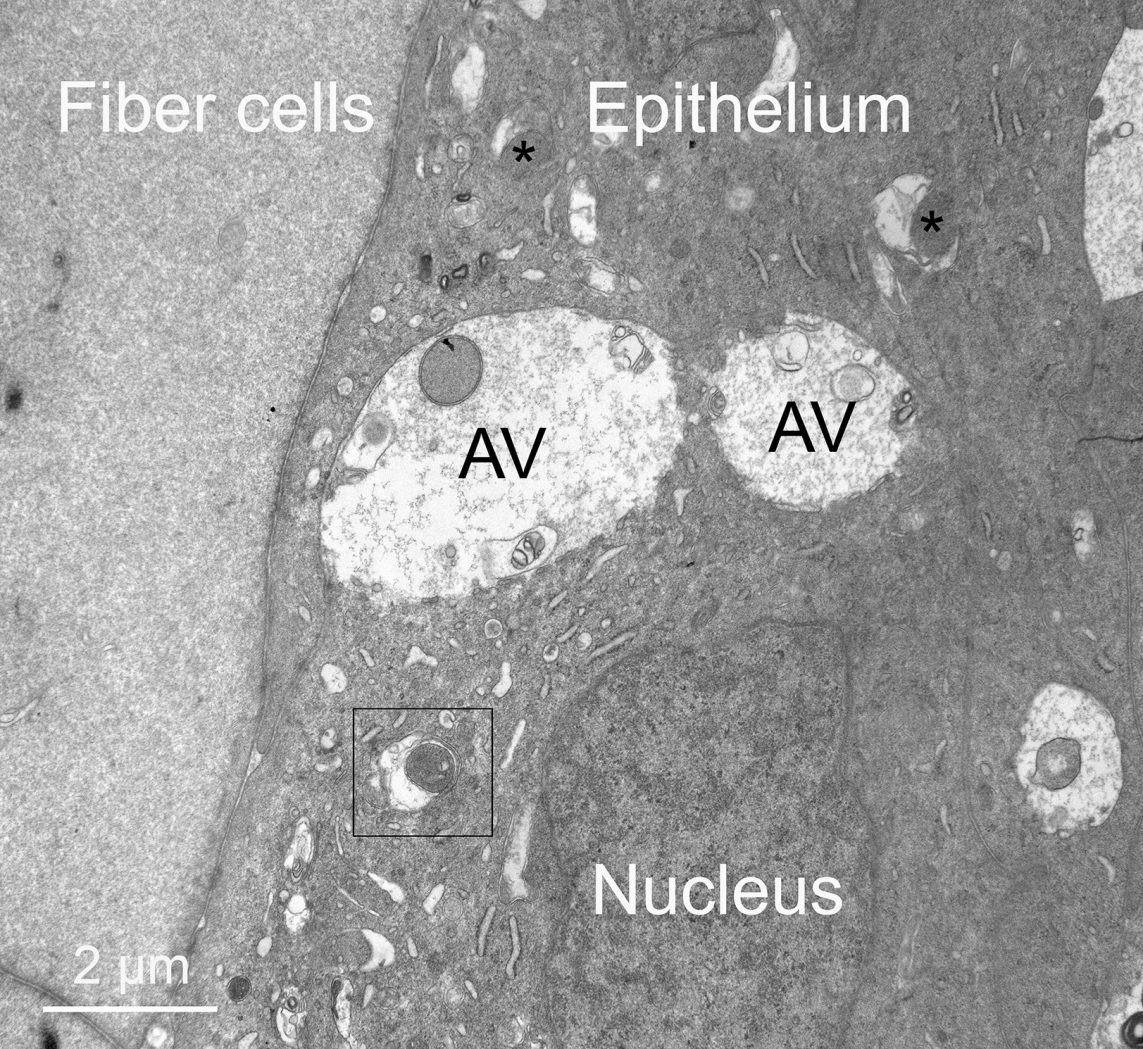
Four-membrane structures within the lens epithelium. Overview of the epithelium-fiber cell interface showing a nucleus, autophagic vesicles (AV) and other characteristic organelles, in addition to unusual oval or circular dense patches (asterisks and boxed region). This image is important for distinguishing young fiber cells from terminal epithelial cells.

**Fig 8:**
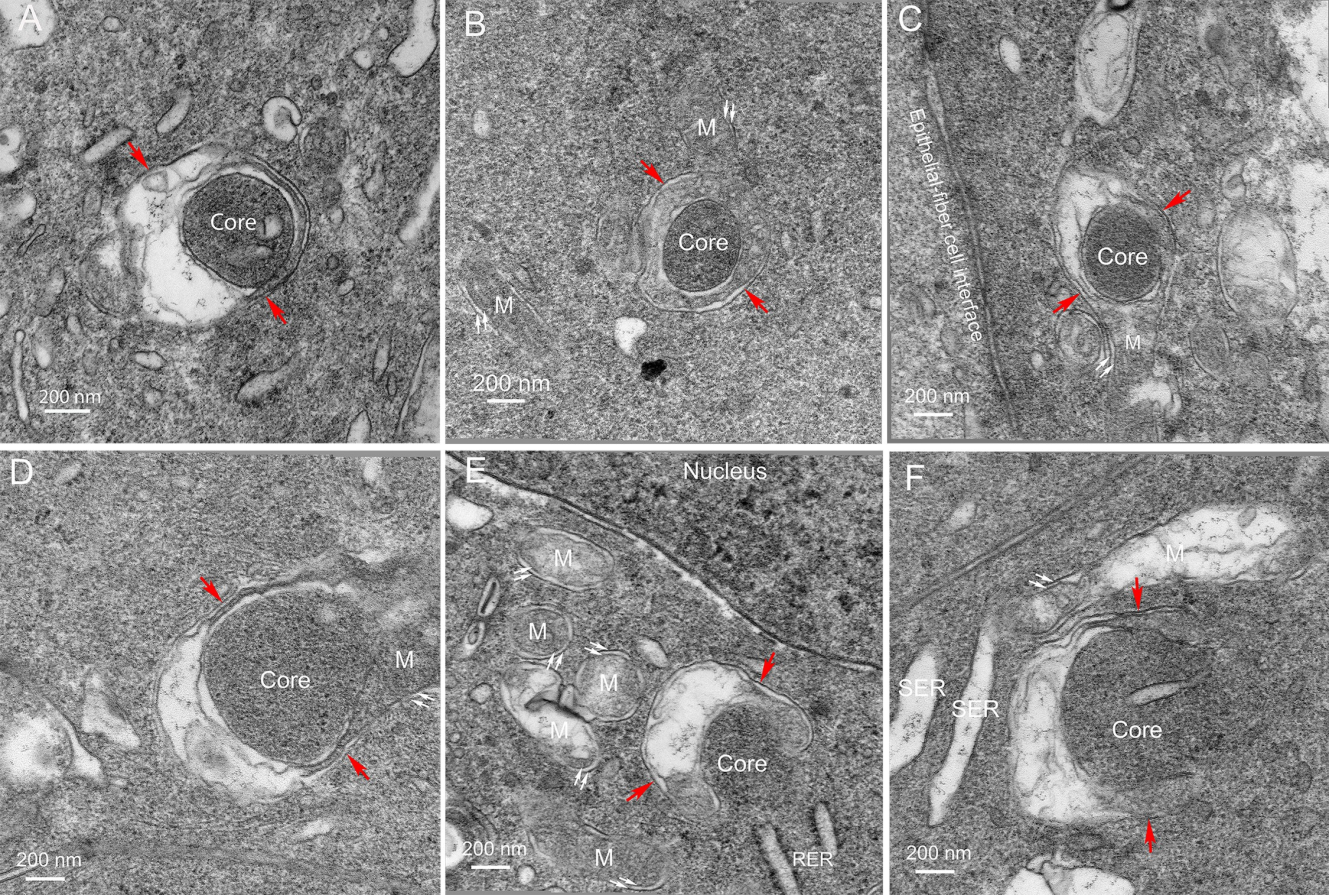
Four-membrane structures within the lens epithelium involve mitochondria. (A) Enlargement of the boxed region in Fig 7 shows a dense circular cluster (core) surrounded by a single membrane and an outer limiting membrane (red arrows). Between these single membranes is a pair of membranes that is irregular in staining and separation distance. This appears to be the initial stages of the formation of a four-membrane structure where it is necessary to cross four membranes to move from the core to the cytoplasm of the epithelial cell. (B) Other variations of the four-membrane structure are visible, including where the irregular paired membranes are very similar to mitochondrial cristae. Although the single membrane around the core and the outer limiting membrane (red arrows) are separated, they are similar to each other. This pattern is easily distinguished from mitochondria (M) with variable cristae and a typical outer double membrane pair (double white arrows). (C) This example at the epithelium-fiber cell interface suggests an association of a mitochondrion (M) with the core in which the mitochondrial cristae appear to flow or migrate around the core. The outer mitochondrial membrane (white arrow pair) is continuous with the single outer limiting membrane (red arrows) whereas the single membrane around the core (clearly visible in two-thirds of the circumference) is separated from the irregular membrane pair that on either side of the core is continuous with the cristae from the labelled mitochondria. (D) A core has a similar relation to a mitochondrion (M; paired white arrows) as in (C) with the presumed cristae extending around the core. On both sides, the cristae membranes appear to come close together (separated by about 35 nm) for a similar length of about 200 nm in a configuration that many mitochondria display in the equatorial epithelium when the thin section has an optimal orientation. Both regions appear to open into a disordered membrane region bounded in part by the outer limiting membrane (red arrows). (E) In the early stage of formation of a four-membrane structure, the dense core appears to be associated with one side of a curved mitochondria that is wrapping around the core. The association suggests that the core membrane may be derived from an outer mitochondrial membrane and, on the opposite side, the outer limiting membrane (red arrows) may also be derived from an outer mitochondria membrane. In this view, the core is exposed directly to the epithelial cytoplasm with some rough ER (RER) in the vicinity, suggesting these as possible sources of components of the core. In the image are numerous mitochondria (M) with double membrane outer layers (paired white arrows) suggesting that proliferation of mitochondria is characteristic of this region of the equatorial epithelium. (F) A curved and modified mitochondrion appears to wrap around a core with the cristae at the top condensing into closely paired membranes having about 35 nm spacing and 200 nm length, as in (D) above, then opening into disorganized cristae. An adjacent mitochondrion (M) with a double membrane outer layer (white arrows) also has similarly distorted cristae even though it is not involved in formation of a four-membrane structure. This observation suggests that mitochondria in general may be releasing cristae and matrix components into the cytoplasm that could diffuse into the core. In this view the core contains a short segment of smooth ER similar to larger segments (SER) characterized by a single membrane with irregular low density protein-like contents.

The linear structures with the unique four-membrane profile appear to be the active component of the NE in Galago lenses. The distinctive profile and the unique core, which has high staining density and extreme uniformity, are features that permit exploration for similar structures within all regions of the outer cortex. The high staining density of the core facilitated the identification of precursors of the linear structures within lens epithelium adjacent to young fiber cells (Fig 7). The epithelial-fiber cell interface provides an ideal setting to distinguish unique features of the epithelium. The most obvious features other than the expected nucleus are the numerous autophagic vesicles (Fig 7, AV). These are frequently relatively large (over 2 μm diameter), some with characteristic double membrane outer profiles and irregular patterns of cellular components in various stages of degradation. In this overview at low magnification, it is possible to identify several dense aggregates that are potentially NE cores (Fig 7, asterisks and boxed region). High magnification of the boxed region reveals a dark staining cluster (similar to condensed protein) that is labelled as a potential core, adjacent to a pair of irregular membranes and an outer membrane (Fig 8A, red arrows). The core region is surrounded by a single membrane and, together with the paired membranes, confirms that movement from the core to the cytoplasm of this epithelial cell requires crossing four membranes. Therefore, the right side of this object has the configuration of the four-membrane structures in the fiber cell cytoplasm, whereas the left side has disordered membranes that appear to converge onto the irregular pair of membranes above and below the presumptive core. The high quality of cellular preservation in this region is demonstrated by the normal appearance of segments of smooth and rough ER near the upper red arrow.

Several additional examples (Fig 8B–8F) are presented to support the hypothesis that the Galago epithelium contains structures with the four-membrane configuration and involve mitochondria in their formation. Within the cytoplasm of an epithelial cell, where other typical organelles, such as mitochondria, are present (Fig 8B and 8M), a dark core with a single membrane is found surrounded by an irregular pair of membranes and a single outer membrane (Fig 8B, red arrows). In another example adjacent to the epithelial-fiber cell interface (Fig 8C), the core appears to be engulfed by a modified form of the inner mitochondrial cristae membranes of a partially fused mitochondrion (Fig 8C and 8M). The image in Fig 8D raises the possibility that, once a core forms, it may interact with mitochondria such that the outer mitochondria membrane contributes to the outer limiting membrane of the four-membrane objects and the mitochondrial cristae contribute to the irregular pair of membranes. A similar pattern is displayed in another example where the core is bordered by two regions of closely opposed membranes that are potentially collapsed mitochondrial cristae (Fig 8D, near red arrows). The membranes are uniformly separated by 35 nm and both regions are about 200 nm in length. The variable densities across the space may be components of the cristae that are selected as spacers without a clear functional role for this distinctive membrane pattern. It is noted that both regions open on the left side and connect to variable disorganized membranes. Additional insight is obtained from an early stage of formation of the four-membrane configuration (Fig 8E). The core is a dense protein cluster with direct exposure to the epithelial cytoplasm, which contains nearby rough ER. Also significant is the observation that the presumptive core is associated with the surface of a mitochondrion with distorted cristae that appears to be wrapping around the core. This implies that the core membrane may be derived from the outer mitochondrial membrane and, if the engulfment of the core continues, that the outer limiting membrane may also be derived from an outer mitochondrial membrane (Fig 8E, red arrows). The importance of mitochondria in the formation process is further emphasized by the large number of mitochondria in this same image, some with modified cristae (Fig 8E and 8M). The last image in this series shows a mitochondrion with modified cristae partially enclosing a presumptive core with the core containing a segment of smooth ER, another potential source of components of the core (Fig 8F, core). Modified cristae are also present in large adjacent mitochondrion (Fig 8F and 8M), which is clearly not involved in formation of a four-membrane object, suggesting that mitochondria may not be just reorganizing their cristae but may also be releasing components of the cristae and matrix that may migrate across the outer mitochondrial membrane to contribute to the core. This hypothesis is further evaluated in the final set of images.

### D. Genesis of the four-membrane component of the Galago NE

**Fig 9:**
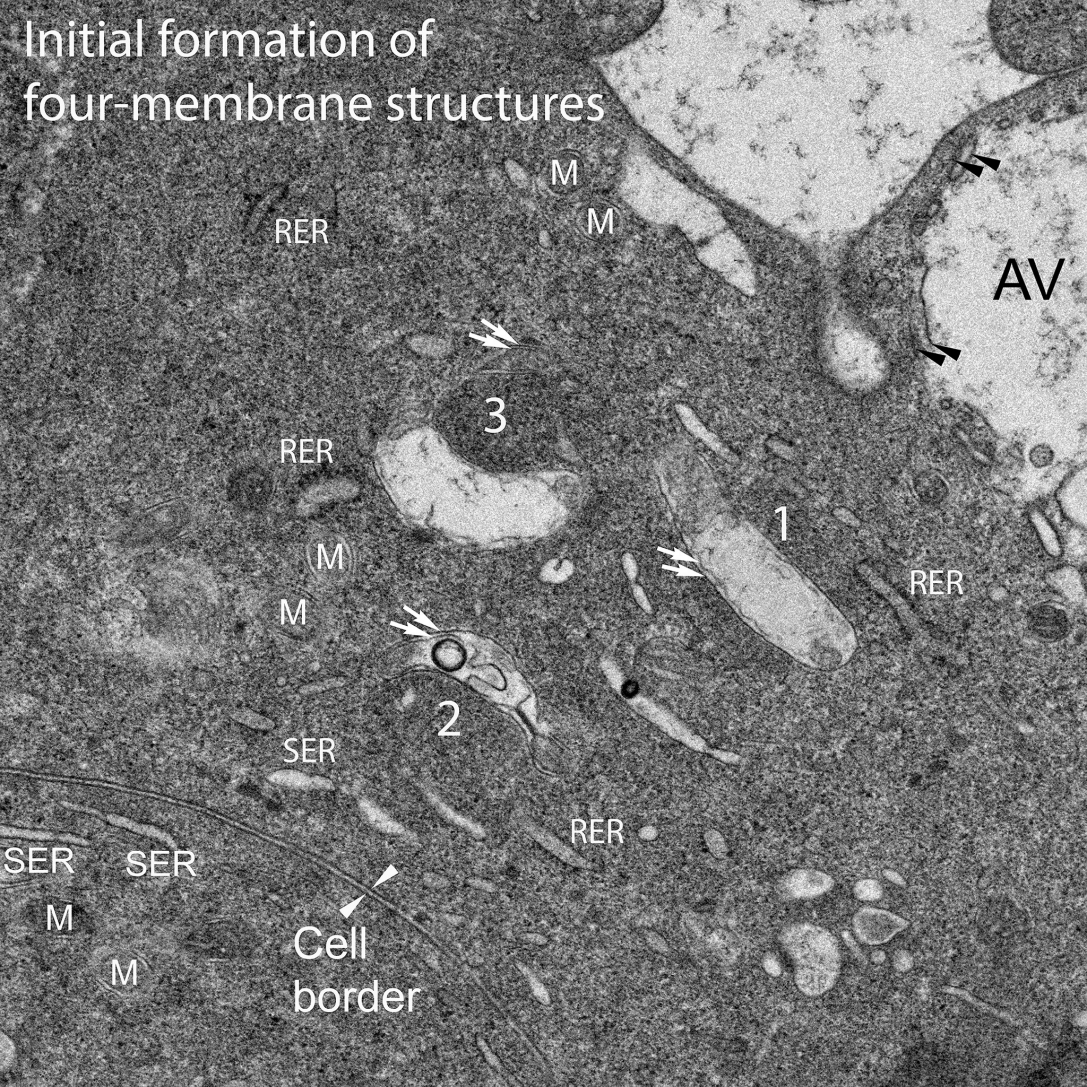
Genesis of the four-membrane structures. The equatorial epithelium of Galago lenses is very active in producing and assembling the components necessary to create the four-membrane objects that are the basis for the NE. A detailed survey of epithelial cells has exposed active regions containing multiple examples of early stages of formation involving modified mitochondria. Three examples here have been placed in logical sequence to illustrate possible mechanisms. An oval of slightly increased staining density, labeled 1, that shows close contact with a long mitochondrion with modified cristae on one end, could represent the initiation of the process. A larger and denser cluster, labeled 2, is associated with a curved mitochondrion that is undergoing typical modifications of cristae including a condensed segment, a disordered region and a circular density that at high magnification contains 5 nm lipid layers. These features and the curvature of the mitochondria around the presumptive core indicate an intermediate state. A still larger and more densely stained circular region, labeled 3, is nearly surrounded by a mitochondrion, including the tip noted with paired white arrows separated from the main body of the modified mitochondria by an oblique section through a nearly intact region. This represents a more advanced stage. Numerous images of these and other stages have been recorded individually (not in clusters) to confirm that they are representative of steps in a continuum of four-membrane structure development, although two-membrane examples predominate here as mitochondria (double arrows for precursors and M for normal mitochondria), black arrowheads for autophagic vesicles (AV) and white arrowheads for the interface between epithelial cells. This cell border corresponds to the uniform narrow space labelled with WGA appearing as thin red lines between epithelial cells in Fig 1. Well-preserved single-membrane organelles, including ER (SER and RER), are also noted.

**Fig 10:**
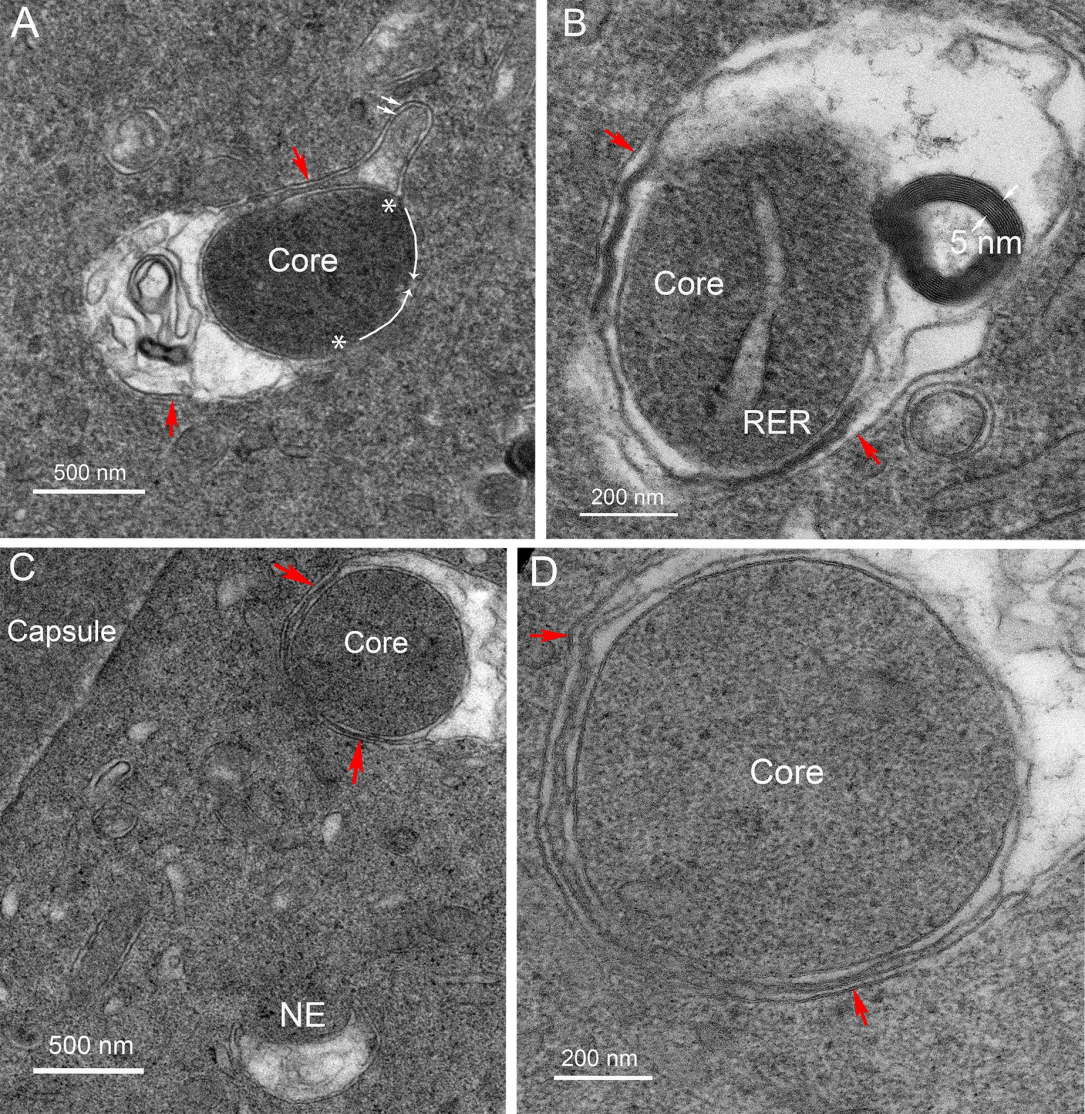
Maturation of the four-membrane structures. (A) Final closure around a core can be envisioned in this example with the extension of the mitochondrion at the asterisks following the path of the white arrows. The outer mitochondrial membrane (paired white arrows) gives rise to the outer limiting membrane (red arrows). (B) In all of the developing four-membrane structures imaged, the mitochondria have extensively modified cristae which may include breakdown and resorbing components as indicated by multilamellar lipids with 5 nm spacing. (C) As the process continues in the epithelium, more advanced stages should be evident, such as the large core with uniform texture and density surrounded by single membrane and a similar membrane as an outermost layer (red arrows). This region must be from the epithelium because of the adjacent capsule and other organelles including a nascent NE. (D) High magnification image confirms the four-membrane configuration and the condensed paired cristae membranes. Remodeling of the disordered cristae membranes to the right further advances the process to more closely resemble the cross-sectional profiles of the linear objects in fiber cell cytoplasm shown in Fig 4.

The genesis of the four-membrane structures in the equatorial epithelium of Galago lenses can be followed from the initial formation of a core as a small dense clustering of protein to the enclosure of a large circular dense uniform array of protein similar to the final core in the observed NE within fully differentiated fiber cells (Figs 9 and 10). Note that all of the changes described occur within the cytoplasm of epithelial cells in the region just prior to the migration of the cells around the fulcrum during the differentiation process (see Fig 1A). A detailed examination of these final epithelial cells reveals high activity regions based on the number, size and configuration of mitochondria. In certain locations it is common to see several initial stages of formation close together as if the signals directing this process are localized. One such region displays three early stages that have been interpreted as a sequence of early genesis of the four-membrane structures (Fig 9, labelled 1–3). Based on the staining and proximity to known organelles, the domain labelled 1 is an oval cluster of protein. The density of the cluster is just barely above background and is effectively imaged by direct viewing at glancing angles. This oval cluster is in contact with the outer mitochondrial membrane of a long cigar-shaped mitochondrion with extensively modified cristae on one end. In the region labelled 2, the cluster is larger and denser, indicating a more advanced stage. Furthermore, the mitochondrion is highly modified in a specific pattern with the nearly intact right end leading into a condensed region of collapsed cristae (about 35 nm thick and 100 nm long; see Fig 8D), and opening into a region of disruption, including a dense ring which at high magnification contains 5 nm lipid bilayers. These modifications suggest that specific rearrangements are taking place as the mitochondrion becomes more closely associated and curves around the early core formation. In the region labelled 3, the core is larger, more circular and more condensed. The associated mitochondrion wraps around the core including the upper portion where the double membrane is clearly visible (Fig 9, paired white arrows). Numerous smooth and rough ER segments are present in this region, as are several mitochondria not involved in four-membrane structure formation.

An example of further enclosing a core by a mitochondrion (Fig 10A) suggests a process for closure. The dense core has a pronounced inner membrane around 2/3 of the perimeter. At contact points with the modified mitochondrion (asterisks), the addition of membrane or movement of the mitochondrial membrane pair along the indicated path (Fig 10A, white arrows) would tend to close the gap and completely engulf the core. After the core formation is initiated, the mitochondrial cristae are remodelled to eliminate material not needed in the final product and to select or conserve whatever degradative components are required for the NE. This apparently involves extensive redistribution and perhaps breakdown of portions of the mitochondrion similar to processes that occur in autophagy, using components within the mitochondria and leaving remnants of the cristae breakdown or remodelling process (Fig 10B). Multilamellar lipids are frequently observed as part of this remodelling process (Fig 10B, 5 nm) that are probably recycled, as suggested for mitophagy [6], as well as for nuclear degradation by the NE in chick embryo lenses [9]. It is noted here that this process of NE formation is distinct from mitophagy because no classical autophagic vesicles are present at any stage within the NE forming structures, although autophagic vesicles are numerous within the adjacent epithelium (see Fig 7).

As this final clearing of excess membranes and other non-essential membrane components proceeds, the prediction is that more mature forms of the four-membrane structures will be observed (Fig 10C). In this example, the presence of a large core with uniform density similar to that found in the final state within differentiated fiber cells (see Fig 4) supports the genesis of these structures within the epithelium and transfer to young fiber cells around the fulcrum. This nearly complete NE core is clearly within the epithelium based on the presence of the capsule and other organelles seen at low magnification (Fig 10C). At high magnification, the core and its single membrane cover are clear, as well as the collapsed cristae membranes and the outer limiting membrane similar to the NE structures in fiber cells (Fig 10D, red arrows). This is sufficient visual evidence to conclude that this four-membrane structure has only to refine and simplify the disordered membranes on one side to reach its final state.

## Discussion

**Fig 11:**
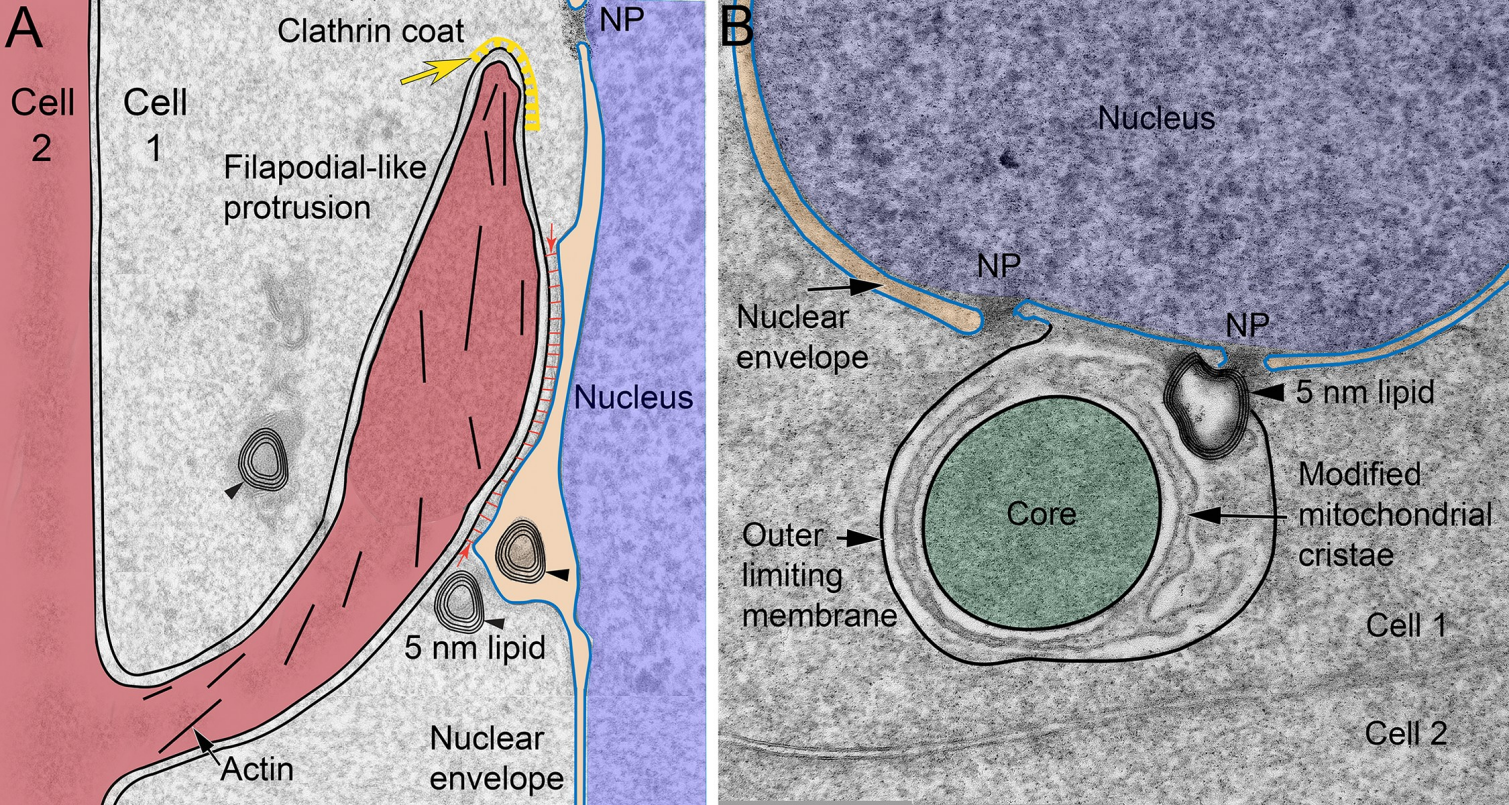
Unique structural features of chicken embryo and Galago adult lens Nuclear Excisosomes. (A) In chick embryo lenses [9], a filopodial-like protrusion from Cell 2 into Cell 1, initiated with a clathrin coat (yellow arrow) and propelled by an internal actin network (short black lines), contacts an outer nuclear envelope membrane to initiate its degradation near the organelle-free zone (OFZ). The contact site distorts the contour of the outer nuclear membrane with close contact (red arrows) and densities across the space similar to cellular junctions. Modeled after Fig 16 in ref [9] with emphasis on the cytoplasmic extension (red) from an adjacent cell and the release of lipid as 5 nm stacks (arrowheads) typical of membrane breakdown in the lens. These protein poor bilayer clusters migrate across the cytoplasm to fuse with the plasma membrane as the OFZ develops. (B) Galago lenses generate a four-membrane structure in the equatorial epithelium with a pronounced core (green) surrounded by a uniform membrane and a similar outer limiting membrane (black line) that fuses with the outer nuclear envelope membranes (blue lines) to initiate degradation. It is hypothesized that the irregular membrane pair between the similar limiting membranes is derived from modified mitochondrial cristae (displayed as in the original image in Fig 5B) and release the degradative enzymes that separate lipid and protein of the nuclear envelope. This process can be initiated at or near nuclear pore complexes (NP), although the main goal is to release lipid in the 5 nm bilayers that build up as the nuclear envelope degrades. An important conclusion from this comparison is that different species can employ distinct structural components to form the Nuclear Excisosome with the similar goal of nuclear envelope degradation as a key final step in OFZ formation. Preliminary results from higher primate species reinforce this conclusion, because they employ different structural components in their Nuclear Excisosomes than displayed in this figure.

The discovery of the unique cellular components in the NE of chick embryo lenses provided a framework for the exploration of analogous structures in Galago lenses. We sought linear projections from membrane surfaces that contacted nuclei near the OFZ and contained a core of actin microfilaments [9]. No such structures in Galago lenses could be identified, although numerous typical ball-and-socket interlocking devices with dark staining actin cores were observed, as are common in mammalian lenses (see Figs 1B, 3B and 4A). Numerous unique linear structures within fiber cell cytoplasm, not showing any clear association with neighbouring cells, were observed in confocal images using dyes that labeled lipid bilayers within membranes (see Fig 2). These linear structures had a number of unusual properties. They were numerous enough to be present in each fiber cell, even young cells next to the epithelium, suggesting that they were formed long before they were used in nuclear degradation near the OFZ (see Fig 2A). The linear objects in confocal images were consistently about 1 μm diameter with lengths up to 30 μm showing increased association with nuclei progressively through the cortex from the epithelium up to the OFZ where they disappeared along with the nuclei (see Fig 2). These features are consistent with their role in nuclear degradation. Additional unique features characterized the internal ultrastructure in TEM images. The cross-sectional profiles consistently contain a central core that stains as dense homogeneous packed protein surrounded by a single bilayer membrane of uniform width (see Fig 4A and 4B). The outer limiting membrane is similar in appearance with uniform width and staining. The space between these two nearly identical membranes is occupied by a pair of membranes that are non-uniform in staining and separation (see Fig 4A and 4B). This generates a four-membrane structure that is unlike any organelle in the lens (or any other tissue to our knowledge). Strong evidence for the four-membrane structures to be a component of the Galago NE and involved in nuclear degradation came from images showing an initial direct interaction with degrading nuclei in which the outer limiting membrane appeared to fuse with outer nuclear envelope membrane (see Figs 5 and 6). The initial contact at or near nuclear pore complexes released lipid components in the form of 5 nm bilayers (see Figs 3C, 5 and 6) and modified protein in an expanded outer limiting membrane loosely enclosing the appendages on degrading nuclei (see Figs 3 and 6). Many of the NE appendages contained remnants of the distinctive core of dense homogeneous packed protein (see Figs 3, 5 and 6). The unique cell biological features of the Nuclear Excisosome from chick embryo lenses [9] and Galago (bush baby) adult lenses described here are summarized in Fig 11.

The unusual core and four-membrane profile of the linear structures were employed as a guide to establish that similar structures could be found in the Galago epithelium near the equatorial plane (see Fig 7). At high magnification, it was observed that mitochondria were involved in the initial formation of these unique structures in regions that showed a proliferation of mitochondria with significantly modified cristae (see Fig 8). An early modification of mitochondria appeared to be on the outer surface, which collected a cluster of proteins of unknown source or composition (see Fig 9). The proteins could migrate from the cytoplasm, from ER or from modified mitochondria through the outer mitochondrial membrane (see Figs 8–10). Whatever the source, once the core protein cluster began to form, a mitochondrion appeared to wrap around the core as one step in enclosing the core in an outer mitochondrial membrane (see Figs 8–10). Modified cristae formed the irregular pair of membranes and the entire structure was enclosed in another outer mitochondrial membrane (see red arrows in Figs 8 and 10). This pattern of formation was consistent with the final four-membrane linear structures in which the inner and outer membranes were similar to each other and indistinguishable from mitochondrial outer membranes that are rich in porin channels with beta-barrel configurations that have uniform thickness about 7 nm [20, 21]. The irregular staining and spacing of the paired membranes was consistent with modified mitochondrial cristae membranes [22, 23] that can contain many integral membrane protein complexes with large extensions from the membrane surface (including electron transport chain, F_0_F_1_-ATPsynthase, TIM translocation complexes and proteases; [24]) and can be readily distinguished from membrane pairs in other organelles. The genesis of the four-membrane structures in the equatorial epithelium was consistent with more advanced stages that are similar to the final four-membrane linear structure in the fully differentiated fiber cell cytoplasm (see Fig 10). It is hypothesized that once the modified cristae membranes are refined, the mature forms remain in the cytoplasm and migrate around the fulcrum into nascent fiber cells. This hypothesis is consistent with the presence of the four-membrane linear structures in the youngest fiber cells and the absence of any evidence for generation of the structures from within fiber cells. Additional evidence is needed to understand how the initial globular formations can elongate or fuse into the linear structures within fiber cell cytoplasm and how these structures can be stabilized until they are triggered to interact with the nuclear envelope to begin its degradation.

The mechanism of degradation used by the Galago NE may be specific to properties of the mitochondria used to form these unique structures. Although the exact identity of the degradative molecules employed is unknown, it is interesting to consider the possibility that the four-membrane structures are assembled to provide a compartment for the modified cristae containing degradative enzymes, such as the AFG3L2 and YMEL1 AAA+ proteases, known to reside in the inner mitochondrial membrane and influence many protein quality control functions [25, 26]. These unusual four-membrane assemblies and modifications of mitochondria are not observed in epithelia of other lenses recently examined using the same preparation techniques, including chick embryos, humans and macaque monkeys [9] (Costello MJ, et al., Invest Ophthalmol Vis Sci. 2017;58:ARVO E-Abstract 1213) and were not found in the Galago anterior epithelium, suggesting that the generation of the NE in the equatorial epithelium is a property of the differentiation program specific to Galago (and perhaps related species) and not of epithelia in general.

In this preliminary study, the emphasis has been on the initiation of the final stage of OFZ formation involving the removal of fiber cell nuclei. Details of the later stages of this denucleation process, captured in part by the confocal image in Fig 2D showing nuclear fragmentation, condensation and final removal at the OFZ, are important and will be presented with comparisons with higher primates in future studies. This approach is taken in part because of the extensive and unusual modifications of mitochondria needed to create the four-membrane linear structures that are the active components of the Galago NE. Such morphological reorganizations of mitochondria have not been reported previously, including studies of giant mitochondria [27–29], mitochondrial networks [30] and pathological states [31]. Even modern imaging methods [32] have not revealed any modifications of normal mitochondrial cristae [33] that are similar to those seen in the formation of the four-membrane structures. Once the NE begins to degrade the Galago nuclear envelope, it is clear that the process is coordinated with the removal or reduction of nucleoplasm because nuclear volume decreases along with indentations and other shape changes without evident release of large portions of the nuclear content. Furthermore, this process in Galago begins in the presence of detectable nuclear lamina based on confocal images (see Fig 2C and 2D where nuclei are surrounded by a thin bright rim indicating an intact nuclear envelope) and direct imaging in TEM (see Fig 6B). These observations are in contrast to the reports for mouse where the nuclear lamina is removed early in the denucleation process to allow entry of lysosomes containing hydrolytic enzymes [34, 35]. Because small single-walled vesicles (potentially containing enzymes) are not seen here or in chick embryo lenses [9], it is likely that the mechanism for mouse is different. A portion of this work was presented at an international eye research meeting (Costello MJ, et al., Invest Ophthalmol Vis Sci. 2019;60:ARVO E-Abstract 2233).

## Conclusions

The linear four-membrane structures in the fiber cell cytoplasm near the OFZ are the active component of the Galago NE that initiates the degradation of the nuclear envelope. The origin of these unique structures is from modified mitochondria in the equatorial epithelium. Although the maturation of these structures and their assembly into the linear structures has not been observed directly, it is logical to suggest that they appear within the fiber cell cytoplasm of the youngest fiber cells that have migrated around the fulcrum during the final transition of epithelial cells to fiber cells. These properties clearly distinguish the NE of Galago lenses from those of chick embryo lenses and support the conclusion that different species may have individual pathways for the final removal of organelles to generate the transparent OFZ found in the cores of all vertebrate lenses. These observations also point to the identification of a novel function for mitochondria in NE formation and lens fiber cell differentiation.
